# Investigating alignment-free machine learning methods for HIV-1 subtype classification

**DOI:** 10.1093/bioadv/vbae108

**Published:** 2024-07-29

**Authors:** Kaitlyn E Wade, Lianghong Chen, Chutong Deng, Gen Zhou, Pingzhao Hu

**Affiliations:** Department of Computer Science, University of Western Ontario, London, ON N6A 3K7, Canada; Department of Computer Science, University of Western Ontario, London, ON N6A 3K7, Canada; Department of Computer Science, University of Western Ontario, London, ON N6A 3K7, Canada; Department of Computer Science, University of Western Ontario, London, ON N6A 3K7, Canada; Department of Computer Science, University of Western Ontario, London, ON N6A 3K7, Canada; Department of Biochemistry, University of Western Ontario, London, ON N6A 3K7, Canada

## Abstract

**Motivation:**

Many viruses are organized into taxonomies of subtypes based on their genetic similarities. For human immunodeficiency virus 1 (HIV-1), subtype classification plays a crucial role in infection management. Sequence alignment-based methods for subtype classification are impractical for large datasets because they are costly and time-consuming. Alignment-free methods involve creating numerical representations for genetic sequences and applying statistical or machine learning methods. Despite their high overall accuracy, existing models perform poorly on less common subtypes. Furthermore, there is limited work investigating the impact of sequence vectorization methods, in particular natural language-inspired embedding methods, on HIV-1 subtype classification.

**Results:**

We present a comprehensive analysis of sequence vectorization methods across machine learning methods. We report a *k*-mer-based XGBoost model with a balanced accuracy of 0.84, indicating that it has good overall performance for both common and uncommon HIV-1 subtypes. We also report a Word2Vec-based support vector machine that achieves promising results on precision and balanced accuracy. Our study sheds light on the effect of sequence vectorization methods on HIV-1 subtype classification and suggests that natural language-inspired encoding methods show promise. Our results could help to develop improved HIV-1 subtype classification methods, leading to improved individual patient outcomes, and the development of subtype-specific treatments.

**Availability and implementation:**

Source code is available at https://www.github.com/kwade4/HIV_Subtypes

## 1 Introduction

Human immunodeficiency virus 1 (HIV-1) is a global public health concern with over 39 million active cases worldwide as of 2023 (World Health Organization 2023). HIV-1 has a high degree of genetic variability due to its high mutation rates (Cuevas *et al.* 2015, Adhiambo *et al.* 2021), leading to varying degrees of pathogenicity and drug resistance (Taylor *et al.* 2008, Nastri *et al.* 2023). HIV-1 subtype classification or subtyping, refers to the categorization of HIV-1 into distinct taxonomic group based on genetic similarity.

HIV-1 isolates are divided into 4 main groups: M, N, O, and P, with group M being the most prevalent (Taylor *et al.* 2008). Group M is further subdivided into 9 pure subtypes: A, B, C, D, F, G, H, J, and K, and over 100 circulating recombinant forms (CRFs), which are the results of recombination events between pure subtypes (Kuiken *et al.* 2003). The most common HIV-1 subtypes are C, A, CRF 01_AE, and B (Serwin *et al.* 2021). Subtype C is by far the most common and accounts for nearly half of all global HIV-1 infections (Williams *et al.* 2023). Subtype B accounts for only 9% of infections worldwide but is responsible for 56% of infections in North America, South America, Western Europe, and Central Europe, leading to overrepresentation in HIV-1 research and online databases (Williams *et al.* 2023).

Within HIV-1 subtypes, genetic variation ranges from 15% to 20%, while variation between subtypes can be as much as 35% (Hemelaar *et al.* 2006). Furthermore, genetic differences in HIV-1 subtypes lead to different clinical manifestations due to variations in pathogenicity, disease progression, and susceptibility to treatments (Nastri *et al.* 2023). In HIV-1, rates of disease progression vary significantly among subtypes (Robertson *et al.* 2000), making subtype classification a crucial step in infection management (Clumeck *et al.* 2008, Hirsch *et al.* 2008). In addition, there are ongoing efforts to develop vaccines and treatment options designed to target-specific HIV-1 subtypes (Elangovan *et al.* 2021). Thus, HIV-1 subtype classification is a crucial and challenging problem in the field of virology.

Traditional methods for HIV-1 subtype classification use sequence alignment-based methods, which involve aligning input or query genetic sequences with curated subtype reference sequences and comparing homologous nucleotide patterns or motifs (Foley *et al.* 2018). Since many alignment-based methods involve computing similarity statistics over a sliding window (Rozanov *et al.* 2004, Pineda-Peña *et al.* 2013), these approaches can be very computationally expensive, making them impractical for long sequences and large datasets. Alignment-based classification methods may also perform poorly on highly variable regions of the genome (Solis-Reyes *et al.* 2018). Furthermore, there can be reproducibility issues with these approaches because they rely on *ad hoc* parameter settings for handling gaps and mismatches, as well as manually curated reference sequences.

Due to these limitations, various alignment-free HIV-1 subtyping methods have been developed. Alignment-free methods involve creating feature vectors, or numerical representations of genetic sequences, and applying statistical or machine learning models. Kameris (Solis-Reyes *et al.* 2018) is an alignment-free HIV-1 subtyping method that uses a *k*-mer sequence vectorization method and classifies HIV-1 subtypes using a support vector machine (SVM) with linear and polynomial kernels. Although Kameris achieves high overall accuracy, it has poor recall across minority classes. Kevolve also uses *k*-mer encoding, but extracts a minimum feature set and uses an ensemble learning method based on SVMs (Lebatteux and Diallo 2021). Although Kevolve achieves good classification performance, it tends to mistakenly classify recombinant subtypes as pure subtypes. Others (Tang *et al.* 2021) use a *k*-mer and position-based vectorization method in conjunction with multi-class k-nearest neighbours (KNN) algorithm that uses a majority vote. This approach has nearly perfect HIV-1 subtype classification performance, however, this model was developed using only a single gene, so the results may not generalize well to the full-length HIV-1 genome. Furthermore, previous work lacks reproducibility as the software developed is no longer maintained and the datasets and specific implementation details are often unavailable.

Although there has been much work investigating sequence vectorization methods, there has been limited work comparing sequence vectorization methods across machine learning models. Furthermore, many representation methods are based on sequence statistics such as *k*-mer frequency, nucleotide distribution, and average position and the application of natural language-inspired vectorization methods has yet to be explored for HIV-1 subtype classification. Thus, we aim to develop an improved method for HIV-1 subtype classification. We compare the performance of existing sequence vectorization methods across a variety of machine learning models. We also explore the effect of two natural language-inspired embeddings, Word2Vec and Word2Vec with Term Frequency-Inverse Document Frequency (TF-IDF), and investigate their impact on HIV-1 classification.

## 2 Methods

**Figure 1.**
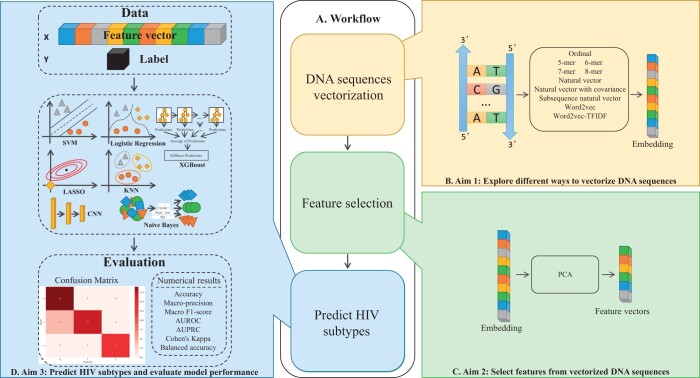
Outline of the proposed research. (A) Overview of the workflow. (B) Sequence vectorization methods. (C) Dimensionality reduction using principal component analysis. (D) Classifying HIV-1 subtypes and evaluating model performance.

We aim to explore 10 different sequence vectorization methods for HIV-1 subtype classification using 7 different machine learning and deep learning models (Fig. 1).

### 2.1 Dataset and preprocessing

We obtained 20 110 full-length HIV-1 genome sequences from the Los Alamos National Laboratory (LANL) HIV Sequence Database (Kuiken *et al.* 2003), representing 289 HIV-1 subtypes. We omit any unknown sequences to ensure our data is labelled. Using a threshold of 18, as used in previous work (Solis-Reyes *et al.* 2018), we discard subtypes containing 18 or fewer examples. The resulting dataset comprised 15 018 sequences from 28 different subtypes, of which, 19 are recombinant subtypes and 9 are pure subtypes. Subtype counts range from 19 to 9806 samples. Subtype B comprises over 65% of our dataset and this overrepresentation is consistent with the existing Euro-centric bias in HIV-1 research (Williams *et al.* 2023). Subtypes C, CRF 01_AE, and A are among the most common subtypes in our dataset and comprise 12.7%, 7.9%, and 3.5%, of the dataset, respectively. This reflects the real-world prevalence of these subtypes. For further details about the HIV-1 subtypes used in our study, see Supplementary Table 1.

### 2.2 Sequence embedding and vectorization

Unaligned genomic sequences have variable lengths and in order for these sequences to be used in machine learning models, they must first be vectorized to create feature vectors of equal length. In our study, we explore ordinal, *k*-mer, natural vector, and natural language-based encoding methods.

#### 2.2.1 Ordinal encoding

Ordinal encoding, which represents our baseline encoding, involves representing each of the four nucleotides (A, T, C, and G) as a number between 0 and 1. For example, A is encoded as 0.25, T as 0.50, C as 0.75, and G as 1.00. To ensure all feature vectors are of equal length, zeroes are appended to the beginning of each feature vector. While one-hot encoding is more standard for categorical genetic data, classical machine learning methods generally do not accept one-hot encoded data due to its sparsity and the additional channel dimension it introduces.

#### 2.2.2 k-mer

*k*-mers are *k* length substrings contained within a biological sequence, and are commonly used for representing and comparing biological sequences (Blaisdell 1986). Generating *k*-mer feature vectors involves counting the frequency of all substrings of length *k*. For genetic sequences composed of 4 nucleotides, there are $4^{k}$ possible *k*-mers. We generate all possible *k*-mers of length 5, 6, 7, and 8 and determine the frequency of each *k*-mer to create feature vectors of size 1024, 4096, 16 384, and 65 536. Our choice of *k* is based on previous HIV-1 subtype classification studies that use *k *=* *6 (Solis-Reyes *et al.* 2018) and *k *=* *8 (Ma *et al.* 2020). We normalize all feature vectors by the length of the sequence, ensuring that the representations are invariant to sequence length.

#### 2.2.3 Natural vector

The natural vector sequence encoding method (Deng *et al.* 2011) creates a 12D feature vector that incorporates nucleotide frequency and sequence-wide position information for each nucleotide (Huang *et al.* 2014). Let $S=(s_{1},s_{2},\ldots s_{n})$ be a nucleotide sequence of length *n* and let M={A,C,G,T}. For m∈M, let the indicator function $w_{m}(\cdot ):M\to \{0,1\}$ be defined as:

(1) $$w_{m}(s_{i})=\{\begin{array}{cc} 1, & \;\text{if}\;s_{i}=m,\\ 0, & \text{otherwise} \end{array}$$

such that $s_{i}\in M$ and i=1,2,…,n. Let the count of each nucleotide *m* in *S* be:

(2) $$n_{m}=\sum_{i=1}^{n}w_{m}(s_{i})$$

The average location (*μ*) of nucleotide *m* in sequence *S* is:

(3) $$\mu_{m}=\sum_{i=1}^{n}i\frac{w_{m}(s_{i})}{n_{m}}$$

The second central moment of position (*D*_2_) for nucleotide *m* in *S* is given by:

(4) $$D_{2}^{m}=\sum_{i=1}^{n}\frac{{\left(i-\mu_{m}\right)}^{2}w_{m}(s_{i})}{n_{m}n}$$

Thus, the 12D natural vector is defined as follows:

$$\left(n_{A},n_{T},n_{C},n_{G},\mu_{A},\mu_{C},\mu_{G},\mu_{T},D_{2}^{A},D_{2}^{C},D_{2}^{G},D_{2}^{T}\right)$$

After computing the 12D natural vector, we normalize each component by the sequence length.

#### 2.2.4 Natural vector with covariance

Since the traditional 12D natural vector representation only considers the distribution of each nucleotide in isolation, this method cannot account for relationships between pairs of nucleotides (Sun *et al.* 2022). To address this, six additional terms representing the pairwise covariance of nucleotides can be added to the natural vector representation. In this method, the indicator function $w_{ml}(\cdot ):M\to \{0,1\}$, where l,m∈M, is defined as follows:

(5) $$w_{ml}(s_{i})=w_{lm}(s_{i})=\{\begin{array}{cc} 1, & \;\text{if}\;s_{i}=m\;\text{or}\;l,\\ 0, & \text{otherwise} \end{array}$$

The covariance between nucleotides *m* and *l* is given by:

(6) $$\mathrm{Cov}(m,l)=\sum_{i=1}^{n}\frac{\left(i-\mu_{m})(i-\mu_{l})w_{ml}(s_{i}\right)}{n\sqrt{n_{m}}\sqrt{n_{l}}}$$

This gives the following 18D natural vector representation that includes pairwise covariance:

$$\begin{array}{l} (n_{A},n_{T},n_{C},n_{G},\mu_{A},\mu_{C},\mu_{G},\mu_{T},D_{2}^{A},D_{2}^{C},D_{2}^{G},D_{2}^{T},\\ Cov(A,C),\mathrm{Cov}(A,G),\mathrm{Cov}(A,T),\mathrm{Cov}(C,G),\\ \mathrm{Cov}(C,T),\mathrm{Cov}(G,T)) \end{array}$$

After computing the 18D natural vector with covariance terms, we normalize each component by the sequence length.

#### 2.2.5 Subsequence natural vector

The 12D and 18D natural vector representations capture only global nucleotide distributions. However, since nucleotides are not distributed equally across the HIV-1 genome (de Lima-Stein *et al.* 2014), these representation may be insufficient. Thus, to capture local nucleotide distributions, we use the subsequence natural vector representation method (He *et al.* 2020).

In this method, the sequence is divided into *P* nonoverlapping segments or subsequences. We select the value for *P* as 130 using the following equation (He *et al.* 2020):

(7) P=⌊H/(12* log(H))⌋

where *H* is the number of HIV-1 samples in our dataset. We then compute the natural vector for each subsequence, as defined above. After concatenating the natural vectors for each subsequence, we are left with 1560D feature vectors.

#### 2.2.6 Word2Vec

Word2Vec (Mikolov *et al.* 2013) is generally used to map natural language vocabularies to high-dimensional vector spaces with a core idea of capturing semantic relationships between words by analysing their distribution patterns in context. This model learns the representation of each word as a fixed-length vector through observing its co-occurrence patterns within the context window. Word2Vec has two main architectures: Skip-gram, which predicts the context words given a target word, and continuous bag of words (CBOW), which predicts the target word using the context words.

**Figure 2.**
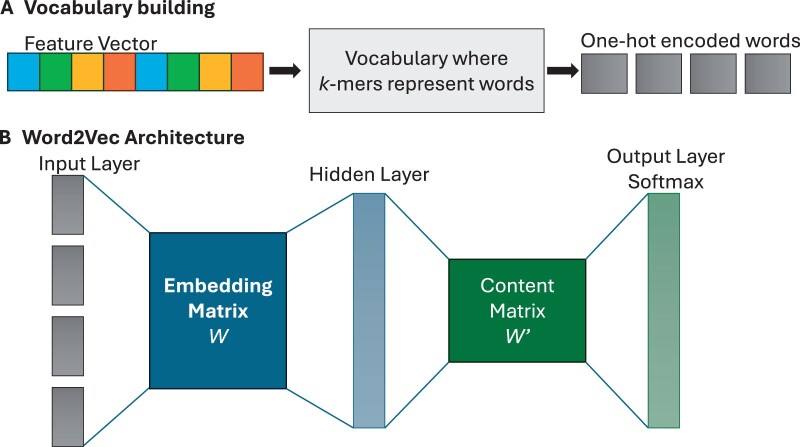
Overview of Word2Vec. (A) To create the vocabulary for Word2Vec, each HIV-1 sequence (feature vector) is divided into *k*-mers, which represents words. Each word is then encoded using one-hot encoding. (B) The Word2Vec architecture consists of three layers—the input layer, the hidden layer, and the output layer, which involves applying Softmax.

Our Word2Vec model is based on the CBOW architecture and has 3 layers (Fig. 2). The first is the input layer, denoted as *z*, which is a one-hot encoded representation of context words; the second is the hidden layer denoted by *h*, and is obtained by multiplying the input layer *z* with a weight matrix *W*:

(8) $$h=W^{T}z$$

The third layer is the output layer, where a score *u_j_* is computed for each target, followed by the application of the Softmax function to obtain the posterior distribution of the target words. This layer is represented as:

(9) $$u_{j}={\left(v_{w_{j}}^{\prime }\right)}^{T}h$$

where $v_{w_{j}}^{\prime }$ is the *j*-th column of the weight matrix for hidden layer to output;

(10) $$\begin{array}{c} p(w_{j}|w_{I})=y_{j}=\frac{\exp(u_{j})}{\sum_{j^{\prime }=1}^{V}exp(u_{j^{\prime }})}\\ =\frac{\exp((v_{w_{j}}^{\prime }{)}^{T}v_{w_{I}})}{\sum_{j^{\prime }=1}^{V}exp((v_{w_{j^{\prime }}}^{\prime }{)}^{T}v_{w_{I}})} \end{array}$$

where *y_j_* is the output of the *j*-th unit in the output layer, $p(w_{j}|w_{I})$ represents the conditional probability of the target word *w_j_* given the context word *w_I_*, *I* represents the index position of the context word, and *V* is the size of the vocabulary. During training, the model predicts the surrounding context words for each target word. After training, the weights of the hidden layer contain the learned word embeddings. We trained the Word2Vec model with the CBOW architecture to learn the patterns of *k*-mer tokens in our HIV-1 genome sequences. We used a sliding window of length *k* with a step size of 1 to produce overlapping *k*-mers. We then took the average vector of those *k*-mers in each sentence. In our study we explore *k*-mer tokens of size 5, 6, 7, and 8, and vectors ranging from 50 to 300 dimensions.

#### 2.2.7 Word2Vec with TF-IDF

Despite the simplicity and effectiveness of the original Word2Vec method, it still overlooks the impact of vocabulary frequency on the importance of different words. Therefore, we introduced the TF-IDF into our Word2Vec method to weigh the *k*-mers. The core idea of TF-IDF is that if a word or phrase has a high frequency (TF) in a sentence and occurs rarely in other sentences, it is considered to have good discriminatory power, making it suitable for classifying that sentence (Dang *et al.* 2020). We utilized scikit-learn tools to generate the TF-IDF values for each word, and then calculated the average vector of each sentence with words multiplied by their respective TF-IDF weights. As with Word2Vec, we explored using 5-, 6-, 7-, and 8-mers as words and feature vectors ranging from 50 to 300 dimensions.

### 2.3 Dimensionality reduction: principal component analysis

Principal component analysis (PCA) (Pearson 1901) is a widely used statistical method for reducing dimensionality. It transforms the original features into a new set of orthogonal components, ordered by the variance they explain. In our study, we use a threshold of 90% for the cumulative explained variance ratio (Supplementary Table 2). For subsequence natural vector and the 5-, 6-, and 7-mer encodings, we train our models with and without PCA. We perform an ablation analysis to assess the effect of PCA on classification (Supplementary Table 3). Due to the high-dimensionality and sparsity of the ordinal and 8-mer encodings, we opt to train our model using PCA rather than without it. Since natural vector, natural vector with covariance, Word2Vec, and Word2Vec with TF-IDF have lower dimensionality, we do not apply PCA.

### 2.4 Random oversampling

After applying PCA, we split our data into training (80%) and testing (20%) sets that are stratified by subtype. To address the imbalance in our dataset, we use random oversampling (Lemaître *et al.* 2017), in which examples from minority classes are randomly duplicated, to create a desired minority class size. Although random oversampling is able to achieve good performance in empirical studies (Batista *et al.* 2004), there can be generalization issues that arise from duplicating data. To mitigate the possibility of overfitting, we use a conservative oversampling strategy in which minority class sizes are tripled.

### 2.5 Subtype classification

Our approach encompasses an analysis of both classical machine learning and deep learning techniques for HIV-1 subtype classification. Each model is trained on preprocessed feature vectors and for each model and we tune hyperparameters using RandomizedSearchCV from scikit-learn (version 1.3.2) using the suggested parameter ranges in the documentation (Pedregosa *et al.* 2011). We set the number of parameter combinations to 10, use 5-fold cross-validation, and use accuracy as the scoring metric. The best-performing model is then used to classify HIV-1 subtypes in both the training and testing datasets. For a detailed overview of each machine learning method, please refer to Supplementary Section 1.

#### 2.5.1 Multi-class logistic regression

The core of logistic regression (LR) is to model the probabilities of different classes based on input features using a logistic function (Cox 1958). Our multi-class LR model is based on scikit-learn’s Multinomial LR framework, which utilizes the Softmax function (Pedregosa *et al.* 2011) to predict probabilities and cross-entropy loss for training. The hyperparameter search for our multi-class LR model explores values of the inverse regularization parameter ranging from 0.01 to 10, considers the ‘newton-cg’, ‘saga’, and ‘sag’ solver algorithms, and varies the number of iterations from 100 and 10 000.

#### 2.5.2 eXtreme Gradient Boosting

eXtreme Gradient Boosting (XGBoost) (Chen and Guestrin 2016) is a powerful and efficient implementation of gradient boosting algorithms. Since it can effectively capture complex nonlinear patterns in data, it performs well in multi-class classification tasks using biological data (Chen *et al.* 2019). XGBoost uses gradient-boosted decision trees as base learners, which are built sequentially. Each new tree corrects the errors made in previous iterations, thereby improving the model’s accuracy step by step. This iterative approach, combined with regularization options, makes XGBoost an efficient model that is well-suited to multi-class classification tasks.

Our model is based on the framework defined in the XGBoost (version 2.0.2) Python package (Chen and Guestrin 2016) using a subsample ratio of 0.5 and a column subsample ratio for each tree of 0.5. In the hyperparameter search, we vary the learning rate from 0.01 to 0.3, the maximum depth from 1 to 10, the number of estimators from 10 to 200, and the minimum loss reduction from 0 to 2.

#### 2.5.3 Least Absolute Shrinkage and Selection Operator

Least Absolute Shrinkage and Selection Operator (LASSO) (Tibshirani 1996) is frequently used to analyse high-dimensional datasets because it introduces a regularization term to the loss function and encourages simpler models with fewer parameters. These characteristics are particularly beneficial in the context of high-dimensional genetic data and help to prevent overfitting and enhance model interpretability.

Our model is based on the multinomial LR framework from scikit-learn (Pedregosa *et al.* 2011) and uses a ℓ1 penalty with the ‘saga’ solver. For hyperparameter tuning, the value of the inverse regularization coefficient (C) ranges from 0.01 to 5, and the maximum number of iterations ranges from 100 to 500.

#### 2.5.4 Naive Bayes

Due to its simplicity, the Naive Bayes classifier (John and Langley 1995) is also a popular option for multi-class classification tasks. The classifier is based on Bayes’ theorem and operates under the assumption that the features in the dataset are independent of each other. Although this assumption is naive, the model is able to handle high-dimensional genetic data and has performed well on bioinformatics tasks such as classifying virus proteins (Feng *et al.* 2013). For our model, we use scikit-learn’s Gaussian Naive Bayes framework (Pedregosa *et al.* 2011) and for hyperparameter tuning, the value for variance smoothing varies between 1e−8 and 1e−10.

#### 2.5.5 K-nearest neighbours

The KNN algorithm (Fix and Hodges 1985) is widely used in multi-task classification tasks. The core of the KNN model involves classifying each data point based on the majority label of its closest neighbours in the feature space. KNN has two key parameters: The number of neighbours (*K*) and the distance metric used for identifying neighbours. During training, the model identifies KNNs based on the distance metric and the classification is performed by a majority vote among these *K* neighbours. The class that appears most frequently within this subset is assigned to the data point.

To build our model, we use scikit-learn’s KNeighborsClassifier (Pedregosa *et al.* 2011), which uses the KNNs vote algorithm. The hyperparameter search explores values between 1 and 30 for the number of neighbours, Euclidean and Manhattan distance metrics, and ‘uniform’ and ‘distance’ weight functions. Using the ‘uniform’ setting, all points in a neighbourhood are weighted equally, while ‘distance’ weighs points based on the inverse distance.

#### 2.5.6 Support vector machine

SVMs (Cortes and Vapnik 1995) are commonly used for classification tasks and involve finding a hyperplane that best separates classes in feature space. Although SVMs are binary classifiers, their functionalities can be extended to multi-class tasks using the One-versus-Rest (OvR) strategy. The OvR approach involves training multiple binary classifiers to differentiate one class from all remaining classes. Using this approach, the decision function is computed for each classifier, and the class corresponding to the classifier with the highest decision function value is chosen as the output.

Using scikit-learn (Pedregosa *et al.* 2011), we wrap an SVM model within a OneVsRestClassifier to train one classifier per class. The target class is treated as the positive class, while all other classes form the negative class. The hyperparameter search explores regularization parameter values ranging from 0.1 to 10, linear, polynomial, and radial basis function kernels, and sets gamma values to ‘auto’ or ‘scale’. Using ‘auto’, the value of gamma is 1/num_features, and using ‘scale’, the value is 1/(num_features * *X*.var()), where *X* is the feature matrix and var() is the variance.

#### 2.5.7 1D convolutional neural network

**Figure 3.**
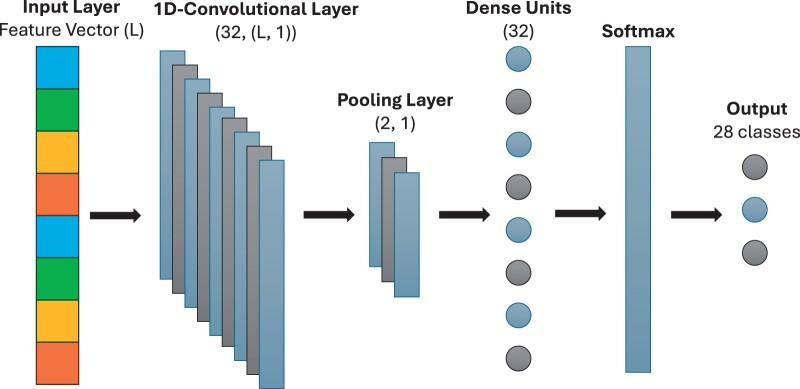
1D-CNN architecture for HIV-1 subtype classification. *L*, length of the feature vector.

One Dimensional Convolutional Neural Networks (1D-CNNs) have shown success for tasks involving sequential data such as genetic data (Zhang *et al.* 2021). Our 1D-CNN architecture is constructed using the Keras framework (Chollet 2015) and begins with a 1D convolutional layer and we specify the number of filters and the kernel size. Each filter in this layer performs convolution operations on the input sequence, which can effectively capture local dependencies. Following the convolutional layer, a max-pooling layer with a pool size of 2 is used to reduce the dimensionality of the data, enhancing the network’s ability to generalize and reducing the computational load. The network then flattens the pooled features and passes them through a dense layer with a specified number of units, each employing a ReLU activation function for nonlinearity. The final layer is a Softmax layer, which can output the probability distribution across the HIV-1 subtypes. Figure 3 outlines the architecture of our network.

The hyperparameter search explores kernel sizes ranging from 2 to 4 and varies the numbers of filters (16, 32, and 64) and dense units (32, 64, and 128). It also explores batch sizes of 8, 10, and 16, epoch sizes of 50, 100, or 150, and considers the ‘adam’ and ‘rmsprop’ optimizers.

### 2.6 Evaluation metrics

To evaluate the performance of our HIV-1 subtype classification models, we consider eight performance metrics: Accuracy, balanced accuracy, precision, recall, F1-score, area under the receiver operating characteristic (AUROC), area under the precision-recall curve (AUPRC), and Cohen’s Kappa. In addition, a confusion matrix is created for each model in order to assess model performance across all 28 classes. All performance metrics are computed using scikit-learn. For a detailed overview of the evaluation metrics, please see Supplementary Section 2.

## 3 Results

### 3.1 Ordinal encoding

Table 1 summarizes the performance of ordinal-based sequence vectorization for HIV-1 subtype classification. Overall, ordinal encoding has poor performance across each machine learning and deep learning model we explored. In particular, the Naive Bayes classifier ranks among the lowest for all performance metrics. This may be because the Gaussian Naive Bayes classifier assumes that all features are independent and that each class follows a Gaussian distribution. Evolutionary constraints in key functional regions of the HIV-1 genome, along with the presence of hypermutation, which introduces nonrandom patterns of nucleotide substitutions, may lead to dependencies among nucleotides (de Lima-Stein *et al.* 2014). Thus, the assumptions of a Gaussian distribution and nucleotide independence may not hold.

**Table 1.** Performance of ordinal encoding for sequence vectorization across machine learning models.

		Performance metrics ^*^						
Method	Model	Accuracy	Balanced accuracy	Precision	F1 score	AUROC	AUPRC	Cohen’s Kappa
	XGBoost	0.86	0.31	0.68	0.39	0.65	0.30	0.69
	Logistic Regression	0.87	**0.42**	0.66	**0.48**	**0.70**	0.34	0.74
	LASSO	**0.88**	0.41	0.63	0.47	**0.70**	0.34	0.75
	Naive Bayes	0.47	0.32	0.34	0.27	0.65	0.18	0.20
	KNN	0.81	0.33	**0.79**	0.42	0.65	0.32	0.53
	SVM	**0.88**	0.38	**0.79**	0.47	0.68	**0.37**	0.74
Ordinal Encoding	CNN	0.86	0.37	0.55	0.43	0.68	0.29	**0.79**

* The highest value for each performance metric is highlighted in bold.

While the values for accuracy, precision, AUROC, and Cohen’s Kappa fall in the range of 0.7 to 0.8 for LR, LASSO, KNN, and SVM, these metrics may be artificially high due to the class imbalance in our dataset. Since subtype B greatly outnumbers the other subtypes, this class may be predicted more frequently than other classes, leading to high overall accuracy. In addition, the high precision scores indicate that the model is likely making accurate predictions for subtype B, but missing most of the minority classes. The poor predictive ability of minority classes is evident from the low balanced accuracy, recall, F1-score, and AUPRC scores. Overall, ordinal encoding yields poor performance.

### 3.2 k-mer encoding

Based on the results of our PCA ablation study (Supplementary Table 3), we find 5-mer and 6-mer encodings perform better without PCA. From the results of Table 2, as the length of *k* increases from 5 to 7, the overall performance of the model improves. However, as *k* increases from 7 to 8, performance drops across all performance metrics, suggesting that the 8-mer encoding is much less effective. This is likely because the 8-mer encoding is very sparse, so it is more difficult for the model to find meaningful patterns.

**Table 2.** Performance of *k*-mer-based encoding methods for sequence vectorization across machine learning models.

		Performance metrics ^*^						
Method	Model	Accuracy	Balanced accuracy	Precision	F1 Score	AUROC	AUPRC	Cohen’s Kappa
5-mer (no PCA)	XGBoost	0.97	0.73	0.88	0.77	0.86	0.68	0.95
	Logistic Regression	**0.98**	0.80	0.88	0.82	0.90	0.74	0.96
	LASSO	**0.98**	0.80	0.89	0.83	0.90	0.76	0.97
	Naive Bayes	0.97	0.72	0.84	0.75	0.86	0.63	0.95
	KNN	0.97	0.74	0.80	0.72	0.87	0.63	0.94
	SVM	**0.98**	0.79	0.89	0.82	0.89	0.73	0.96
	CNN	**0.98**	0.78	0.86	0.80	0.89	0.72	0.96
6-mer (no PCA)	XGBoost	**0.98**	0.75	0.89	0.78	0.87	0.70	0.97
	Logistic Regression	**0.98**	0.80	0.89	0.83	0.90	0.75	0.97
	LASSO	**0.98**	0.80	0.89	0.83	0.90	0.75	0.97
	Naive Bayes	0.91	0.38	0.60	0.41	0.69	0.29	0.82
	KNN	0.97	0.77	0.82	0.76	0.88	0.67	0.95
	SVM	**0.98**	0.79	0.91	0.81	0.89	0.74	0.96
	CNN	**0.98**	0.76	0.84	0.74	0.89	0.71	0.95
7-mer (with PCA)	XGBoost	**0.98**	**0.84**	**0.94**	**0.87**	**0.92**	**0.80**	**0.97**
	Logistic Regression	**0.98**	0.80	0.88	0.82	0.90	0.75	**0.97**
	LASSO	**0.98**	0.78	0.87	0.80	0.89	0.72	0.96
	Naive Bayes	0.75	0.76	0.64	0.64	0.88	0.56	0.59
	KNN	0.88	0.49	0.84	0.59	0.74	0.48	0.73
	SVM	0.97	0.80	0.83	0.80	0.90	0.72	0.95
	CNN	**0.98**	0.77	0.88	0.81	0.89	0.73	0.96
8-mer (with PCA)	XGBoost	0.63	0.03	0.03	0.03	0.50	0.04	0.02
	Logistic Regression	0.52	0.03	0.03	0.03	0.50	0.04	0.02
	LASSO	0.58	0.03	0.03	0.03	0.50	0.04	0.03
	Naive Bayes	0.05	0.02	0.04	0.01	0.49	0.04	0.01
	KNN	0.61	0.04	0.03	0.03	0.50	0.04	0.01
	SVM	0.55	0.03	0.03	0.03	0.48	0.04	0.01
	CNN	0.63	0.02	0.03	0.01	0.50	0.04	0.02

* The highest value for each performance metric is highlighted in bold.

Across the machine learning and deep learning models we explored, the 6-mer encoding generally outperforms the 5-mer encoding, and the 7-mer encoding generally outperforms the 6-mer and 5-mer encodings. In fact, the 7-mer sequence encoding, in combination with XGBoost, outperforms nearly every other *k*-mer-based model, as well as every other vectorization method. Our 7-mer and XGBoost model used a learning rate of 0.196, maximum depth of 5, a gamma value of 0.409, and 134 estimators. 7-mer encoding with XGBoost has very high accuracy (0.98), AUROC (0.92), precision (0.94), and Cohen’s Kappa (0.97), along with good balanced accuracy (0.84), recall (0.84), F1-score (0.87), and AUPRC (0.80), indicating that this combination performs well for both majority and minority classes. This is also evident in its confusion matrix (Supplementary Fig. 1), where the model makes very few mistakes overall, as seen by the diagonal line in the confusion matrix. The model tends to struggle with subtypes that contain few examples, such as subtype A3, which contains only 19 examples. It also tends to misclassify CRF subtypes that originated from the same pure subtypes. For example, the model misclassifies some examples of CRF 31_BC as CRF 07_BC. Both of these recombinant subtypes originate from unique recombination events between subtype B and subtype C (Williams *et al.* 2023). This suggests that the 7-mer sequence encoding may fail to fully capture subtle genetic differences between CRFs that originate from the same pure subtypes.

### 3.3 Natural vector encoding

Across every model we explored, the 12D natural vector and 18D natural vector with covariance encoding methods have similar, yet suboptimal performance (Table 3). This suggests that the covariance terms provide little additional information. These encoding methods have good accuracy, yet poor balanced accuracy, precision, recall, and AUPRC, indicating that like ordinal encoding, these methods are especially sensitive to the imbalance in our data. In addition, the poor classification performance indicates that 12 or 18 dimensions are insufficient for distinguishing between HIV-1 subtypes.

**Table 3.** Performance of natural vector-based encoding methods for sequence vectorization across machine learning models.

		Performance metrics ^*^						
Method	Model	Accuracy	Balanced accuracy	Precision	F1 Score	AUROC	AUPRC	Cohen’s Kappa
	XGBoost	0.88	0.42	0.63	0.48	0.71	0.36	0.76
	Logistic Regression	0.79	0.29	0.29	0.27	0.64	0.19	0.59
	LASSO	0.80	0.30	0.29	0.28	0.64	0.19	0.59
	Naive Bayes	0.67	0.31	0.25	0.26	0.65	0.16	0.41
	KNN	0.88	0.48	0.55	0.49	0.73	0.34	0.77
	SVM	0.84	0.31	0.71	0.39	0.65	0.31	0.63
Natural Vector	CNN	0.83	0.43	0.43	0.40	0.71	0.27	0.68
	XGBoost	0.89	0.42	0.65	0.48	0.71	0.36	0.77
	Logistic Regression	0.80	0.30	0.28	0.28	0.64	0.20	0.59
	LASSO	0.80	0.32	0.29	0.29	0.65	0.21	0.60
	Naive Bayes	0.62	0.32	0.22	0.22	0.65	0.14	0.33
	KNN	0.88	0.48	0.57	0.50	0.74	0.34	0.77
	SVM	0.87	0.48	0.52	0.49	0.74	0.34	0.74
Natural Vector with Covariance	CNN	0.85	0.49	0.45	0.46	0.74	0.30	0.72
	XGBoost	0.95	0.51	0.83	0.59	0.75	0.48	0.90
	Logistic Regression	**0.96**	0.59	0.71	0.63	0.79	0.49	**0.92**
	LASSO	**0.96**	**0.60**	0.71	**0.64**	**0.80**	0.50	**0.92**
	Naive Bayes	0.55	0.44	0.37	0.34	0.71	0.24	0.37
	KNN	0.90	0.51	0.50	0.50	0.75	0.34	0.82
	SVM	0.95	0.57	**0.86**	**0.64**	0.78	**0.51**	0.91
Subsequence Natural Vector	CNN	0.95	0.53	0.71	0.59	0.77	0.45	0.90

* The highest value for each performance metric is highlighted in bold.

The subsequence natural vector encoding outperforms both the 12D and 18D natural vector encoding methods, achieving an accuracy of 0.96, precision of 0.86, AUROC of 0.80, and Cohen’s Kappa of 0.92. In contrast, balanced accuracy and F1-score are ∼0.6, while AURPRC is 0.51. Although the subsequence natural vector encoding method is similar in dimensionality to the 5-mer encoding (Supplementary Table 2), the 5-mer encoding achieves consistently higher performance, indicating that *k*-mer encoding is a superior method.

### 3.4 Natural language encoding

Based on the results of Table 4, the Word2Vec encoding shows promise. We systematically evaluate Word2Vec using *k*-mer tokens of size 5, 6, 7, and 8 and explore feature vectors ranging from 50 to 300 dimensions. Our best-performing Word2Vec encoding uses 6-mers as tokens and a 250D feature vector (Supplementary Table 4). With this encoding, an SVM with a regularization parameter of 2.52, a polynomial kernel, and ‘auto’ gamma values achieves accuracy, AUROC, and Cohen’s Kappa values of 0.90 or greater. It also has a precision value of 0.88, an AUPRC of 0.74, an F1-score of 0.82, and a balanced accuracy of 0.80.

**Table 4.** Performance of natural language-based encoding methods for sequence vectorization across machine learning models.

		Performance metrics ^*^						
Method	Model	Accuracy	Balanced accuracy	Precision	F1 Score	AUROC	AUPRC	Cohen’s Kappa
	XGBoost	0.97	0.64	0.82	0.70	0.82	0.58	0.93
	Logistic Regression	**0.98**	**0.80**	0.83	0.81	**0.90**	0.72	**0.96**
	LASSO	**0.98**	0.78	0.82	0.79	0.89	0.70	0.95
	Naive Bayes	0.60	0.28	0.30	0.20	0.63	0.16	0.94
	KNN	0.97	0.70	0.77	0.71	0.85	0.61	0.94
	SVM	**0.98**	**0.80**	**0.88**	**0.82**	**0.90**	**0.74**	**0.96**
Word2Vec	CNN	0.97	0.75	0.80	0.76	0.87	0.67	0.95
	XGBoost	0.81	0.30	0.31	0.30	0.64	0.16	0.63
	Logistic Regression	0.68	0.10	0.14	0.10	0.54	0.06	0.26
	LASSO	0.67	0.05	0.13	0.05	0.51	0.05	0.10
	Naive Bayes	0.24	0.21	0.19	0.12	0.59	0.08	0.12
	KNN	0.81	0.32	0.31	0.31	0.65	0.16	0.64
	SVM	0.69	0.10	0.13	0.10	0.54	0.06	0.28
Word2Vec with TF-IDF	CNN	0.74	0.20	0.22	0.20	0.59	0.11	0.48

* The highest value for each performance metric is highlighted in bold.

Although these metrics are lower in comparison to the 5-mer, 6-mer, and 7-mer encoding methods across all models, Word2Vec outperforms natural-vector-based methods on nearly all performance metrics, despite. Furthermore, Word2Vec also outperforms the ordinal and Word2Vec with TF-IDF encoding methods. These results show that although Word2Vec was designed for natural language, it is able to capture similarities between genetic sequences. There is, however, room for improvement to further tune Word2Vec in order to attain improved performance on genetic data. Overall, this indicates that Word2Vec has unexplored potential as a sequence encoding method.

In contrast, Word2Vec with TF-IDF achieves poor performance, suggesting that the addition of TF-IDF hinders performance. Although Word2Vec and Word2Vec with TF-IDF consider the occurrences of ‘words’ (*k*-mers), TF-IDF is particularly sensitive to rare words. Rare *k*-mers can be useful when distinguishing between subtypes, but since intrasubtype variability can be as high as 20% in HIV-1 (Hemelaar *et al.* 2006), rare *k*-mers could introduce noise, leading to more classification mistakes.

## 4 Discussion

Out of all the encoding methods we explored, *k*-mer encoding generally outperforms others across metrics and machine learning models, with the notable exception of 8-mer encoding, which achieves the lowest performance. In contrast, 7-mer encoding achieves the highest predictive performance, with the best overall combination being 7-mer with XGBoost. Our *k*-mer and Word2Vec models achieve accuracy scores of ∼98% for LR and SVMs, while Kameris achieves accuracy scores of ∼95% and 97% on these models (Solis-Reyes *et al.* 2018). A recent study achieved accuracy scores of over 99% (Tang *et al.* 2021), however, the dataset used in the study was imbalanced and metrics such as balanced accuracy, which can assess overall model performance are omitted. Since it considers all classes, balanced accuracy may provide a more reliable indication of a model’s overall performance. Although our overall accuracy is slightly lower, our best-performing XGBoost model performs well for majority and minority classes.

However, outside of the *k*-mer encoding methods, Word2Vec with SVM achieves the next highest performance. We also note that both XGBoost and LR have good performance across different sequence vectorization methods. These models may outperform other machine learning and deep learning models because they may more accurately capture complex decision boundaries without overfitting, leading to increased generalizability.

Our results also indicate that the choice of sequence vectorization method is just as important as the choice of machine learning model for HIV-1 subtype classification. Since ordinal encoding is based solely on single nucleotides, it fails to capture local and global motifs that may be unique to HIV-1 subtypes. While natural vector-based methods consider global and local sequence characteristics, these methods are based on summary statistics and may fail to capture subtype-specific motifs, especially in minority classes. *k*-mer encoding and Word2Vec, which consider local sequence composition and relative frequency, have improved performance.

Word2Vec’s moderate performance may be attributed to the fact that it relies on semantic relationships between words in a corpus, an assumption that may not directly translate to genetic sequences. In natural language texts, meaning is conveyed through semantic relationships between adjacent elements. While this may be true to an extent for genetic sequences, there are also intricate patterns of nucleotide interactions across the genome. These regions may exhibit complex interactions and dependencies that are not adequately captured by the vector representations learned by Word2Vec. Despite its success in other bioinformatics applications such as RNASeq clustering (Moussa and Măndoiu 2018), Word2Vec with TF-IDF yields poor performance for HIV-1 subtype classification. Since HIV-1 has high variability within and between subtypes, it may be challenging to classify subtypes solely based on the rarity of particular *k*-mers.

Although the results of our work are promising, our study has some limitations. Despite our efforts to address the imbalance in our dataset through random oversampling, this approach may be insufficient. Further studies could involve exploring more sophisticated oversampling methods such as Synthetic Minority Oversampling Technique (SMOTE) in addition to undersampling strategies such as NearMiss that use a KNN-based approach (Krawczyk 2016). In addition, since our method for hyperparameter tuning relies on a random search, we are not guaranteed to find the optimal set of parameters. Future work could involve using a more through hyperparameter approach such as Grid Search. In addition, given the promise of the Word2Vec, further studies are needed in order to explore Word2Vec in combination with other CNN architectures and other deep learning models.

## 5 Conclusion

Our work presents a comprehensive analysis of sequence vectorization techniques and machine learning models for HIV-1 subtype classification. Based on our findings, the sequence vectorization method and machine learning model chosen are of equal importance for HIV-1 subtype classification. We report a 7-mer encoding method that in combination with XGBoost, achieves high predictive accuracy across majority and minority classes. While *k*-mer encoding methods outperform Word2Vec, the combination of Word2Vec with SVM still shows promise for classifying both minority and majority classes. Our thorough analysis of HIV-1 sequence vectorization methods may pave the way for future HIV-1 subtype classification models that are well-suited to classifying rare and recombinant subtypes, leading to improved patient outcomes and the development of novel subtype-specific drugs and vaccines.

## Supplementary Material

vbae108_Supplementary_Data

## Data Availability

The source code for the sequence vectorization and classification methods used in this study are available in our GitHub repository: https://www.github.com/kwade4/HIV_Subtypes.
