# Supplementary material for: Investigating alignment-free machine learning methods for HIV-1 subtype classification
Source: Bioinform Adv. 2024 Jul 29;4(1):vbae108. doi: 10.1093/bioadv/vbae108 (PMC11371153; doi:10.1093/bioadv/vbae108)
Supplement: vbae108_Supplementary_Data [file vbae108_supplementary_data.pdf]

Investigating Alignment-Free Machine Learning Methods for HIV-1 Subtype Classification

Kaitlyn E. Wade[1], Lianghong Chen[1], Chutong Deng[1], Gen Zhou[1]

and Pingzhao Hu[1,2]

[1]Department of Computer Science, University of Western Ontario, 1151 Richmond Street, N6A 3K7, London, Ontario, Canada

[2]Department of Biochemistry, University of Western Ontario, 1151 Richmond Street, N6A 3K7, London, Ontario, Canada

Supplementary Table 1: Summary Statistics for HIV-1 Subtypes Used in This Study.

Subtype Name	Count	Average Sequence Length (nt)	Average GC Content	Description
CRF 79_0107	42	9096	0.417	Recombinant of CRF 01_AE and CRF 07_BC; found in China
CRF 33_01B	83	8836	0.411	Recombinant of CRF 01_AE and subtype B; found in Southeast Asia
01BC	26	8796	0.409	Recombinant of CRF 01_AE and subtype B; distinct from CRF 33_01B
CRF 01_AE	1183	9046	0.412	Recombinant of subtypes A and E; found in Asia and Africa
CRF 63_02A1	21	2783	0.412	Recombinant of CRF 02_AG and sub-subtype A6; found in Russia
A3	19	8821	0.414	Subtype of subtype A; found in Western Africa
CRF 02_AG	168	8764	0.412	Recombinant of subtypes A and G; found in Asia and Central and Western Africa
CRF 07_BC	97	8871	0.411	Distinct recombinant of subtypes B and C; found in China
CRF 31_BC	45	8854	0.409	Recombinant of subtypes B and C; found in Brazil
CRF 57_BC	26	9228	0.418	Distinct recombinant of subtypes B and C; found in China
CRF 08_BC	35	9094	0.417	Distinct recombinant of subtypes B and C; found in China
CRF 11_cpx	18	8905	0.414	Recombinant of subtypes A, G, J, and CRF 01_AE; found in Central and Western Africa
CRF 140_0107	18	8998	0.417	Recombinant of subtypes CRF 01_AE and CRF 07_BC; found in China
CRF 20_BG	23	8742	0.413	Recombinant of subtypes B and G; found in Cuba
A1	458	8986	0.414	Subtype of subtype A; found in Africa
A1CD	118	9027	0.418	Recombinant of subtypes C, D, and sub-subtype A1
CRF A1CG	24	8950	0.415	Recombinant of subtypes C, G, and sub-subtype A1
CRF 35_AD	134	8927	0.418	Recombinant of sub-subtype A1 and subtype D; found in Afghanistan
A6	54	8769	0.411	Subtype of subtype A; found in Former Soviet Union countries
B	9806	8984	0.416	Pure subtype; most prevalent subtype in North and South America, Europe, and Australia
CRF 64_BC	59	8991	0.415	Recombinant of subtypes B and C; found in China
CRF 47_BF1	107	8879	0.414	Recombinant of subtype B and sub-subtype F1; found in South America
C	1914	8984	0.417	Pure subtype; most prevalent subtype worldwide, especially common in Southern and Eastern Africa and parts of Asia
CRF 10_CD	63	8924	0.416	Recombinant of subtypes C and D; found in Africa
D	104	8841	0.415	Pure subtype; found in Central and Eastern Africa
F1	35	9064	0.420	Subtype of subtype F; found in Central and Western Africa
G	41	8896	0.415	Pure subtype; found in Africa and Central Europe
O	29	9093	0.420	“Outlier” group; found in Central and Western Africa

Supplementary Table 2: Feature Dimensionality Before and After PCA

Vectorization Method	Dimensionality before PCA	Dimensionality after PCA
Ordinal	10414	2513
5-mer	1024	259
6-mer	4096	600
7-mer	16384	923
8-mer	65536	3277
Subsequence Natural Vector	1560	258

Supplementary Table 3: Results of PCA Ablation Study Using *k*-mer and Sub-sequence Natural Vector Encodings.

Encoding	Model	Accuracy	Precision	Balanced Accuracy	F1 Score	AUROC	AUPRC	Cohen's Kappa
5-mer (with PCA)	LG	0.98	0.86	0.78	0.8	0.89	0.71	0.96
	XGBoost	0.97	0.81	0.71	0.73	0.85	0.63	0.95
	Lasso	0.98	0.83	0.76	0.77	0.88	0.68	0.96
	SVM	0.97	0.76	0.77	0.74	0.88	0.63	0.95
	Naïve Bayes	0.83	0.72	0.78	0.71	0.89	0.63	0.73
	KNN	0.89	0.85	0.62	0.68	0.8	0.59	0.77
	CNN	0.98	0.84	0.77	0.79	0.88	0.71	0.96
5-mer (no PCA)	LG	0.98	0.88	0.8	0.82	0.9	0.74	0.97
	XGBoost	0.97	0.87	0.73	0.77	0.86	0.68	0.95
	Lasso	**0.98**	**0.89**	**0.8**	**0.83**	**0.9**	**0.76**	**0.97**
	SVM	0.98	**0.89**	0.79	0.82	0.89	0.73	0.96
	Naïve Bayes	0.97	0.84	0.72	0.75	0.86	0.63	0.95
	KNN	0.97	0.8	0.74	0.72	0.87	0.63	0.94
	CNN	0.98	0.86	0.78	0.8	0.89	0.72	0.96
6-mer (with PCA)	LG	**0.98**	0.86	0.79	0.8	0.89	0.72	0.96
	XGBoost	**0.98**	0.88	0.77	0.8	0.89	0.72	0.96
	Lasso	**0.98**	0.88	**0.8**	0.82	**0.9**	0.74	0.96
	SVM	**0.98**	0.85	0.78	0.79	0.89	0.69	0.95
	Naïve Bayes	0.79	0.7	0.79	0.69	0.89	0.6	0.67
	KNN	0.88	0.84	0.52	0.61	0.75	0.51	0.74
	CNN	**0.98**	0.83	0.79	0.8	0.89	0.72	0.95
6-mer (no PCA)	LG	**0.98**	0.89	**0.8**	**0.83**	**0.9**	**0.75**	**0.97**
	XGBoost	**0.98**	0.89	0.75	0.78	0.87	0.7	0.96
	Lasso	**0.98**	0.89	**0.8**	**0.83**	**0.9**	**0.75**	**0.97**
	SVM	**0.98**	**0.91**	0.79	0.81	0.89	0.74	0.96
	Naïve Bayes	0.91	0.6	0.38	0.41	0.69	0.29	0.82
	KNN	0.97	0.82	0.77	0.76	0.88	0.67	0.95
	CNN	**0.98**	0.84	0.76	0.74	0.89	0.71	0.95
7-mer (with PCA)	LG	**0.98**	0.88	0.8	0.82	0.9	0.75	**0.97**
	XGBoost	**0.98**	**0.94**	**0.84**	**0.87**	**0.92**	**0.8**	**0.97**
	Lasso	**0.98**	0.87	0.78	0.8	0.89	0.72	0.96
	SVM	0.97	0.83	0.8	0.8	0.9	0.72	0.95
	Naïve Bayes	0.75	0.64	0.76	0.64	0.88	0.56	0.59
	KNN	0.88	0.84	0.49	0.59	0.74	0.48	0.73
	CNN	**0.98**	0.88	0.77	0.81	0.89	0.73	0.96

Encoding	Model	Accuracy	Precision	Balanced Accuracy	F1 Score	AUROC	AUPRC	Cohen's Kappa
7-mer (no PCA)	LG	**0.98**	0.91	0.8	0.82	0.9	0.76	0.97
	XGBoost	**0.98**	0.89	0.77	0.8	0.88	0.72	0.96
	Lasso	**0.98**	0.89	0.8	0.8	0.91	0.79	0.96
	SVM	**0.98**	0.92	0.76	0.8	0.88	0.72	0.96
	Naïve Bayes	0.89	0.52	0.26	0.32	0.63	0.25	0.76
	KNN	0.96	0.81	0.75	0.72	0.88	0.62	0.93
	CNN	**0.98**	0.87	0.77	0.8	0.86	0.72	0.96
Subsequence Natural Vector (with PCA)	LG	0.92	0.63	0.53	0.56	0.76	0.40	0.86
	XGBoost	0.91	0.8	0.41	0.50	0.70	0.38	0.82
	Lasso	0.92	0.58	0.51	0.52	0.75	0.37	0.85
	SVM	0.91	0.43	0.48	0.43	0.74	0.27	0.83
	Naïve Bayes	0.76	0.45	0.49	0.44	0.74	0.29	0.58
	KNN	0.92	0.55	0.53	0.54	0.76	0.37	0.84
	CNN	0.91	0.58	0.45	0.49	0.72	0.34	0.83
Subsequence Natural Vector (no PCA)	LG	**0.96**	0.71	0.59	0.63	0.79	0.49	**0.92**
	XGBoost	0.95	0.83	0.51	0.59	0.75	0.48	0.90
	Lasso	**0.96**	0.71	**0.60**	**0.64**	**0.80**	0.50	**0.92**
	SVM	0.95	**0.86**	0.57	**0.64**	0.78	**0.51**	0.91
	Naïve Bayes	0.55	0.37	0.44	0.34	0.71	0.24	0.37
	KNN	0.90	0.50	0.51	0.50	0.75	0.34	0.82
	CNN	0.95	0.71	0.53	0.59	0.77	0.45	0.90

Supplementary Table 4: Performance of Word2Vec-Based Encoding Techniques Across Machine Learning Models with Varying *k*-mer Token and Vector Sizes.

Encoding	Model	Accuracy	Precision	Balanced Accuracy	F1 Score	AUROC	AUPRC	Cohen's Kappa
5-mer tokens, 50 dimensions	LG	0.95	0.7	0.71	0.7	0.85	0.58	0.91
	XGBoost	0.95	0.73	0.56	0.61	0.78	0.49	0.91
	Lasso	0.96	0.7	0.71	0.7	0.85	0.58	0.92
	SVM	0.97	0.81	0.71	0.74	0.86	0.65	0.94
	Naïve Bayes	0.85	0.55	0.66	0.57	0.83	0.45	0.73
	KNN	0.95	0.75	0.65	0.66	0.82	0.55	0.9
	CNN	0.95	0.71	0.67	0.68	0.83	0.55	0.91
5-mer tokens, 100 dimensions	LG	0.97	0.8	0.59	0.73	0.75	0.63	0.94
	XGBoost	0.96	0.8	0.59	0.65	0.79	0.55	0.92
	Lasso	0.93	0.59	0.44	0.46	0.72	0.36	0.87
	SVM	0.95	0.71	0.64	0.66	0.82	0.54	0.91
	Naïve Bayes	0.73	0.44	0.44	0.39	0.71	0.3	0.55
	KNN	0.95	0.7	0.66	0.66	0.82	0.54	0.91
	CNN	0.95	0.73	0.66	0.67	0.83	0.54	0.91
5-mer tokens, 150 dimensions	LG	0.97	0.79	0.76	0.77	0.88	0.66	0.94
	XGBoost	0.95	0.8	0.57	0.64	0.78	0.52	0.91
	Lasso	0.97	0.79	0.76	0.77	0.88	0.67	0.95
	SVM	0.98	0.81	0.76	0.78	0.88	0.68	0.95
	Naïve Bayes	0.61	0.21	0.3	0.18	0.64	0.13	0.33
	KNN	0.95	0.7	0.63	0.64	0.81	0.51	0.9
	CNN	0.96	0.77	0.69	0.72	0.84	0.59	0.93
5-mer tokens, 200 dimensions	LG	0.97	0.79	0.75	0.76	0.87	0.66	0.95
	XGBoost	0.96	0.77	0.56	0.62	0.78	0.51	0.92
	Lasso	0.97	0.77	0.74	0.75	0.87	0.64	0.94
	SVM	0.98	0.83	0.77	0.79	0.88	0.7	0.95
	Naïve Bayes	0.59	0.28	0.28	0.18	0.63	0.14	0.33
	KNN	0.96	0.72	0.67	0.68	0.83	0.55	0.92
	CNN	0.97	0.8	0.73	0.75	0.87	0.63	0.94
5-mer tokens, 250 dimensions	LG	0.97	0.84	0.78	0.79	0.89	0.69	0.95
	XGBoost	0.96	0.83	0.61	0.68	0.81	0.56	0.93
	Lasso	0.97	0.82	0.77	0.78	0.88	0.66	0.95
	SVM	0.98	0.85	0.76	0.79	0.88	0.69	0.96
	Naïve Bayes	0.6	0.34	0.31	0.21	0.65	0.17	0.35
	KNN	0.96	0.73	0.62	0.64	0.81	0.52	0.92
	CNN	0.97	0.78	0.7	0.72	0.85	0.6	0.94

Encoding	Model	Accuracy	Precision	Balanced Accuracy	F1 Score	AUROC	AUPRC	Cohen's Kappa
5-mer tokens, 300 dimensions	LG	0.97	0.82	0.77	0.78	0.88	0.67	0.95
	XGBoost	0.96	0.8	0.63	0.63	0.82	0.56	0.93
	Lasso	0.97	0.81	0.76	0.77	0.88	0.67	0.95
	SVM	0.98	0.82	0.75	0.77	0.88	0.69	0.96
	Naïve Bayes	0.54	0.26	0.29	0.16	0.63	0.14	0.28
	KNN	0.96	0.72	0.62	0.64	0.81	0.53	0.92
	CNN	0.97	0.83	0.71	0.74	0.86	0.65	0.95
6-mer tokens, 50 dimensions	LG	0.95	0.7	0.73	0.71	0.86	0.58	0.91
	XGBoost	0.96	0.78	0.6	0.66	0.8	0.54	0.92
	Lasso	0.95	0.7	0.72	0.71	0.86	0.59	0.91
	SVM	0.97	0.82	0.74	0.76	0.87	0.67	0.94
	Naïve Bayes	0.8	0.46	0.54	0.44	0.76	0.32	0.66
	KNN	0.96	0.77	0.65	0.67	0.82	0.56	0.92
	CNN	0.96	0.75	0.67	0.7	0.84	0.59	0.92
6-mer tokens, 100 dimensions	LG	0.97	0.75	0.74	0.74	0.87	0.63	0.93
	XGBoost	0.96	0.85	0.61	0.67	0.8	0.54	0.92
	Lasso	0.97	0.75	0.75	0.75	0.87	0.63	0.94
	SVM	0.98	0.82	0.75	0.77	0.87	0.68	0.96
	Naïve Bayes	0.7	0.4	0.42	0.31	0.7	0.23	0.49
	KNN	0.96	0.76	0.68	0.69	0.84	0.58	0.93
	CNN	0.97	0.75	0.7	0.72	0.85	0.6	0.94
6-mer tokens, 150 dimensions	LG	0.97	0.78	0.76	0.76	0.88	0.66	0.95
	XGBoost	0.97	0.85	0.64	0.71	0.82	0.6	0.93
	Lasso	0.97	0.78	0.75	0.76	0.87	0.65	0.95
	SVM	0.98	0.87	0.76	0.79	0.88	0.7	0.95
	Naïve Bayes	0.7	0.43	0.41	0.32	0.7	0.23	0.49
	KNN	0.97	0.78	0.7	0.71	0.85	0.61	0.94
	CNN	0.97	0.75	0.7	0.72	0.85	0.61	0.94
6-mer tokens, 200 dimensions	LG	0.97	0.76	0.76	0.75	0.88	0.66	0.95
	XGBoost	0.96	0.77	0.59	0.64	0.8	0.53	0.92
	Lasso	0.97	0.76	0.76	0.75	0.88	0.65	0.95
	SVM	0.98	0.85	0.77	0.79	0.89	0.7	0.96
	Naïve Bayes	0.69	0.42	0.4	0.31	0.69	0.23	0.48
	KNN	0.97	0.79	0.7	0.72	0.85	0.62	0.93
	CNN	0.97	0.77	0.7	0.72	0.85	0.61	0.94

Encoding	Model	Accuracy	Precision	Balanced Accuracy	F1 Score	AUROC	AUPRC	Cohen's Kappa
6-mer tokens, 250 dimensions	LG	0.98	0.85	0.78	0.8	0.89	0.71	0.96
	XGBoost	0.96	0.8	0.58	0.64	0.79	0.52	0.92
	Lasso	0.98	0.85	0.8	0.81	0.9	0.72	0.96
	SVM	0.98	0.88	0.79	0.82	0.89	0.73	0.96
	Naïve Bayes	0.6	0.32	0.31	0.2	0.65	0.17	0.36
	KNN	0.97	0.77	0.69	0.7	0.85	0.59	0.93
	CNN	0.97	0.78	0.71	0.73	0.86	0.62	0.94
6-mer tokens, 300 dimensions	LG	0.98	0.83	0.8	0.81	0.9	0.72	0.96
	XGBoost	0.97	0.82	0.64	0.7	0.82	0.58	0.93
	Lasso	0.98	0.82	0.78	0.79	0.89	0.7	0.95
	SVM	0.98	0.88	0.8	0.82	0.9	0.74	0.96
	Naïve Bayes	0.6	0.3	0.28	0.2	0.63	0.16	0.33
	KNN	0.97	0.77	0.7	0.71	0.85	0.61	0.94
	CNN	0.97	0.8	0.75	0.76	0.87	0.67	0.95
7-mer tokens, 50 dimensions	LG	0.95	0.69	0.7	0.69	0.85	0.56	0.91
	XGBoost	0.95	0.75	0.56	0.62	0.78	0.49	0.91
	Lasso	0.96	0.7	0.71	0.7	0.86	0.57	0.92
	SVM	0.97	0.77	0.71	0.73	0.85	0.62	0.94
	Naïve Bayes	0.64	0.32	0.33	0.23	0.66	0.17	0.4
	KNN	0.95	0.73	0.65	0.67	0.82	0.54	0.91
	CNN	0.96	0.7	0.65	0.66	0.82	0.52	0.92
7-mer tokens, 100 dimensions	LG	0.97	0.76	0.76	0.76	0.88	0.65	0.94
	XGBoost	0.96	0.85	0.63	0.69	0.81	0.58	0.93
	Lasso	0.97	0.79	0.78	0.78	0.89	0.67	0.94
	SVM	0.98	0.84	0.78	0.8	0.89	0.71	0.96
	Naïve Bayes	0.67	0.32	0.34	0.25	0.67	0.21	0.46
	KNN	0.97	0.78	0.71	0.72	0.85	0.62	0.94
	CNN	0.97	0.78	0.7	0.73	0.85	0.61	0.94
7-mer tokens, 150 dimensions	LG	0.98	0.82	0.77	0.78	0.88	0.68	0.95
	XGBoost	0.96	0.81	0.62	0.67	0.81	0.55	0.93
	Lasso	0.97	0.82	0.78	0.79	0.89	0.69	0.95
	SVM	0.98	0.83	0.76	0.78	0.88	0.7	0.96
	Naïve Bayes	0.66	0.32	0.35	0.25	0.67	0.19	0.43
	KNN	0.97	0.77	0.71	0.72	0.85	0.62	0.94
	CNN	0.97	0.79	0.78	0.78	0.89	0.66	0.95

Encoding	Model	Accuracy	Precision	Balanced Accuracy	F1 Score	AUROC	AUPRC	Cohen's Kappa
7-mer tokens, 200 dimensions	LG	0.98	0.82	0.78	0.79	0.89	0.7	0.96
	XGBoost	0.97	0.84	0.7	0.74	0.85	0.64	0.94
	Lasso	0.98	0.8	0.77	0.78	0.89	0.68	0.95
	SVM	0.98	0.85	0.79	0.81	0.9	0.72	0.96
	Naïve Bayes	0.73	0.47	0.43	0.37	0.71	0.29	0.53
	KNN	0.97	0.81	0.75	0.76	0.87	0.66	0.94
	CNN	0.97	0.84	0.73	0.76	0.86	0.67	0.95
7-mer tokens, 250 dimensions	LG	0.98	0.84	0.77	0.79	0.88	0.7	0.96
	XGBoost	0.97	0.84	0.65	0.7	0.82	0.59	0.94
	Lasso	0.98	0.85	0.78	0.79	0.89	0.7	0.96
	SVM	0.98	0.88	0.78	0.81	0.89	0.72	0.96
	Naïve Bayes	0.73	0.46	0.41	0.35	0.7	0.28	0.54
	KNN	0.97	0.8	0.71	0.73	0.86	0.63	0.94
	CNN	0.98	0.83	0.79	0.8	0.89	0.72	0.96
7-mer tokens, 300 dimensions	LG	0.98	0.83	0.78	0.79	0.89	0.7	0.96
	XGBoost	0.97	0.83	0.69	0.73	0.85	0.64	0.94
	Lasso	0.98	0.82	0.78	0.79	0.89	0.69	0.96
	SVM	0.98	0.85	0.78	0.8	0.89	0.72	0.96
	Naïve Bayes	0.72	0.47	0.44	0.37	0.71	0.29	0.53
	KNN	0.97	0.79	0.68	0.7	0.84	0.6	0.94
	CNN	0.97	0.79	0.74	0.75	0.87	0.64	0.95
8-mer tokens, 50 dimensions	LG	0.96	0.72	0.71	0.71	0.86	0.58	0.92
	XGBoost	0.95	0.74	0.56	0.62	0.78	0.5	0.91
	Lasso	0.96	0.72	0.71	0.7	0.85	0.58	0.92
	SVM	0.97	0.76	0.71	0.72	0.85	0.6	0.94
	Naïve Bayes	0.7	0.35	0.38	0.29	0.68	0.2	0.49
	KNN	0.95	0.73	0.64	0.66	0.82	0.53	0.91
	CNN	0.96	0.76	0.7	0.71	0.85	0.57	0.93
8-mer tokens, 100 dimensions	LG	0.97	0.76	0.75	0.75	0.87	0.65	0.94
	XGBoost	0.96	0.77	0.61	0.66	0.8	0.54	0.93
	Lasso	0.97	0.76	0.76	0.76	0.88	0.65	0.95
	SVM	0.98	0.83	0.77	0.78	0.88	0.69	0.96
	Naïve Bayes	0.74	0.45	0.42	0.36	0.71	0.28	0.56
	KNN	0.97	0.82	0.69	0.71	0.84	0.59	0.93
	CNN	0.97	0.77	0.71	0.73	0.86	0.61	0.93

Encoding	Model	Accuracy	Precision	Balanced Accuracy	F1 Score	AUROC	AUPRC	Cohen's Kappa
8-mer tokens, 150 dimensions	LG	0.98	0.78	0.77	0.77	0.88	0.68	0.95
	XGBoost	0.97	0.84	0.66	0.71	0.83	0.61	0.94
	Lasso	0.97	0.78	0.76	0.76	0.88	0.66	0.95
	SVM	0.98	0.84	0.77	0.78	0.88	0.7	0.96
	Naïve Bayes	0.71	0.42	0.37	0.3	0.68	0.24	0.5
	KNN	0.97	0.78	0.72	0.73	0.86	0.63	0.94
	CNN	0.97	0.74	0.71	0.7	0.85	0.58	0.94
8-mer tokens, 200 dimensions	LG	0.98	0.84	0.8	0.81	9	0.73	0.96
	XGBoost	0.97	0.84	0.64	0.7	0.82	0.59	0.94
	Lasso	0.98	0.84	0.77	0.79	0.89	0.69	0.95
	SVM	0.98	0.87	0.78	0.8	0.89	0.71	0.96
	Naïve Bayes	0.66	0.38	0.36	0.27	0.67	0.22	0.43
	KNN	0.97	0.77	0.69	0.71	0.85	0.6	0.94
	CNN	0.98	0.82	0.75	0.78	0.88	0.68	0.95
8-mer tokens, 250 dimensions	LG	0.98	0.82	0.78	0.79	0.89	0.71	0.96
	XGBoost	0.97	0.8	0.67	0.71	0.83	0.61	0.94
	Lasso	0.98	0.79	0.77	0.78	0.89	0.69	0.95
	SVM	0.98	0.81	0.76	0.76	0.88	0.68	0.95
	Naïve Bayes	0.7	0.35	0.37	0.28	0.68	0.22	0.49
	KNN	0.97	0.77	0.73	0.74	0.86	0.64	0.95
	CNN	0.98	0.83	0.76	0.79	0.88	0.71	0.96
8-mer tokens, 300 dimensions	LG	0.98	0.85	0.79	0.81	0.89	0.72	0.96
	XGBoost	0.97	0.84	0.66	0.71	0.83	0.6	0.94
	Lasso	0.98	0.85	0.79	0.81	0.89	0.72	0.96
	SVM	0.98	0.86	0.78	0.8	0.89	0.71	0.96
	Naïve Bayes	0.69	0.4	0.38	0.3	0.69	0.25	0.47
	KNN	0.97	0.79	0.75	0.75	0.87	0.65	0.94
	CNN	0.98	0.81	0.74	0.76	0.87	0.67	0.96

Supplementary Section 1: Details of the Machine Learning Methods

The following are detailed discussions of the methodology used in this study.

Section 1.1: Multi-class Logistic Regression

The core of logistic regression (LR) is to model the probabilities of different classes based on input features using a logistic function. Our multi-class LR model is based on scikit-learn's Multinomial Logistic Regression framework, which utilizes the Softmax function to predict probabilities and cross-entropy loss for training.

Mathematically, the way the Softmax function generalizes Logistic Regression to multiple classes can be defined as:

$$P(Y = k|X = x) = \frac{e^{x^T \beta_k}}{\sum_{l=1}^{K} e^{x^T \beta_l}} \tag{1}$$

where $P(Y = k|X = x)$ is the probability that class k is the correct classification, x is the feature vector, β_k is the coefficient vector for class k, and K is the total number of classes. The coefficients of each class are learned by maximizing the likelihood of the training data and this process uses optimization algorithms to find coefficients that lead to the best predictions. This function then calculates the probabilities of each class and the class with the highest probability is selected as the output of the model.

Section 1.2: eXtreme Gradient Boosting

eXtreme Gradient Boosting (XGBoost) is a powerful and efficient implementation of gradient boosting algorithms. Since it can effectively capture complex nonlinear patterns in data, it performs well in multi-class classification tasks using biological data. XGBoost uses gradient-boosted decision trees as base learners, which are built sequentially. Each new tree corrects the errors made in previous iterations, thereby improving the model's accuracy step by step.

Mathematically, the XGBoost algorithm updates the model using the formula:

$$y_i^{(t)} = y_i^{(t-1)} + \eta \cdot f_t(x_i) \tag{2}$$

where $y_i^{(t)}$ represents the prediction at the t-th iteration, $y_i^{(t-1)}$ is the prediction from the previous iteration, η is the learning rate, and $f_t(x_i)$ is the output of the new decision tree at iteration t. This iterative approach, combined with regularization options, makes XGBoost an efficient model that is well-suited to multi-class classification tasks.

Section 1.3: Least Absolute Shrinkage and Selection Operator (LASSO)

The LASSO model uses a cost function that includes a penalty proportional to the absolute value of the coefficients, which can be expressed as:

$$\text{Cost Function } = \frac{1}{2n} \sum_{i=1}^{n} (y_i - \sum_{j=1}^{p} x_{ij} \beta_j)^2 + \lambda \sum_{j=1}^{p} |\beta_j| \tag{3}$$

where y_i represents the target variable, x_{ij} are the feature variables, β_j are the coefficients, n is the number of samples, p is the number of features, and λ is the regularization parameter. During the training process, LASSO reduces the coefficients of less important features to zero.

Section 1.4: Naïve Bayes

At their core, Naive Bayes classifiers compute the posterior probability of each class based on the input features using the formula:

$$P(k|\ x) = \frac{P(x \mid k)\ P(k)}{P(x)} \tag{4}$$

where $P(k|\ x)$ denotes the probability of class k given a feature vector x, $P(x \mid k)$ is the likelihood of observing the features x in class k, $P(k)$ is the prior probability of class k, and $P(x)$ is the overall probability of the features.

Section 1.5: K-Nearest Neighbours

The K-Nearest Neighbors (KNN) algorithm is widely used in multi-task classification tasks. The core of the KNN model involves classifying each data point based on the majority label of its closest neighbours in the feature space. KNN has two key parameters: the number of neighbours (K) and the distance metric used for identifying neighbours. During training, the model identifies K nearest neighbours based on the distance metric and the classification is performed by a majority vote among these K neighbours. The class that appears most frequently within this subset is assigned to the data point. In our work, we consider two distance metrics: Euclidean and Manhattan.

The Euclidean distance, also known as straight line distance, between two points (x_1, y_1) and (x_2, y_2) is calculated as:

$$\text{Euclidean Distance} = \sqrt{(x_2 - x_1)^2 + (y_2 - y_1)^2} \tag{5}$$

The Manhattan distance, also known as the city block distance, between two points (x_1, y_1) and (x_2, y_2) is calculated as:

$$Manhattan\ Distance = |x_2 - x_1| + |y_2 - y_1| \tag{6}$$

Section 1.7: Support Vector Machines

Support Vector Machines (SVMs) are commonly used for classification tasks and involve finding a hyperplane that best separates classes in feature space. Mathematically, the decision function for an SVM in the binary classification setting is given by:

$$f(x) = \text{sign}\left(\sum_{i=1}^{N} \alpha_i y_i K(x_i, x) + b\right) \quad (7)$$

where x is the input feature vector, x_i are the support vectors, y_i are the labels of the support vectors, α_i are the learned weights, K is the kernel function, and b is the bias.

Section 1.8: One-dimensional Convolutional Neural networks

One Dimensional Convolutional Neural Networks (1D-CNNs) have shown success for tasks involving sequential data such as genetic data. Our 1D-CNN architecture is constructed using the Keras framework and begins with a 1D convolutional layer and we specify the number of filters and the kernel size. Each filter in this layer performs convolution operations on the input sequence, which can effectively capture local dependencies. The convolution operation is mathematically represented as:

$$O_i = \sum_{j=1}^{K} I_{i+j-1} \cdot W_j \quad (8)$$

where O_i is the output, I is the input sequence, and W_j is the weight of the filter. Following the convolutional layer, a max-pooling layer with a pool size of 2 is used to reduce the dimensionality of the data, enhancing the network's ability to generalize, and reducing the computational load. The network then flattens the pooled features and passes them through a dense layer with a specified number of units, each employing a ReLU activation function for non-linearity. The final layer is a Softmax layer, which can output the probability distribution across the HIV-1 subtypes.

Supplementary Section 2: Details of Performance Metrics

Section 2.1: Accuracy, Balanced Accuracy, Macro-Precision, and Macro-F1-Score

The accuracy of a classification model is the fraction of correctly classified cases out of the total number of predictions. Accuracy provides insight into how well the model is able to classify all subtypes overall. However, for imbalanced datasets, a model may have high accuracy overall, but low accuracy for minority classes. To address this limitation, we also consider balanced accuracy, which is implemented in scikit-learn as the average recall for each class and is equivalent to macro-recall. To gain a deeper understanding of each model's performance, we consider macro-precision and macro-F1 score. Macro-level metrics involve computing an unweighted average, meaning each class is given equal importance, regardless of its prevalence in the dataset. This is particularly useful for unbalanced datasets, where the class distribution is unequal. Precision refers to the model's ability to identify instances of a class (or HIV-1 subtype) correctly, while macro-precision is the unweighted average of precision across classes. Alongside precision, we also consider the F1-score, which is the harmonic mean of precision and recall. Macro-F1 score is the unweighted average for the F1-score across classes. In summary, balanced accuracy, macro-precision and macro-F1 score provide insight into the model's performance for each subtype.

Section 2.2: AUROC

AUROC is a measure of the model's ability to distinguish between positive and negative classes. The receiver operating characteristics (ROC) curve represents a trade-off between true positive and false positives across different threshold values, and the area under this curve represents the model's ability to distinguish between classes. Since HIV-1 subtyping is a multi-class classification task, each class is treated as a binary classification task against the rest (OvR). Macro-AUROC is calculated by computing the AUROC for each class individually and then taking the unweighted average. An AUROC of 0.5 suggests that the model's performance is no better than a random guess.

Section 2.3: AUPRC

AURPC can provide insight into a model's ability to correctly identify positive cases, while minimizing the number of false positives. In other words, it assesses the ability of a model to balance precision and recall. Macro-AURPC involves is calculated by computing the AUPRC for each class individually and taking the unweighted average.

Section 2.4: Cohen’s Kappa

Cohen's Kappa quantifies the agreement between the observed accuracy and the expected accuracy by chance. It measures the agreement between the predictions of a model and the actual classes, while accounting for the possibility of agreement by chance.

Section 2.5: Confusion Matrix

A confusion matrix summarizes the performance of each model by providing a breakdown of the true positive, true negatives, false positives, and false negatives. This gives insight into the particular types of classification errors made for each class.

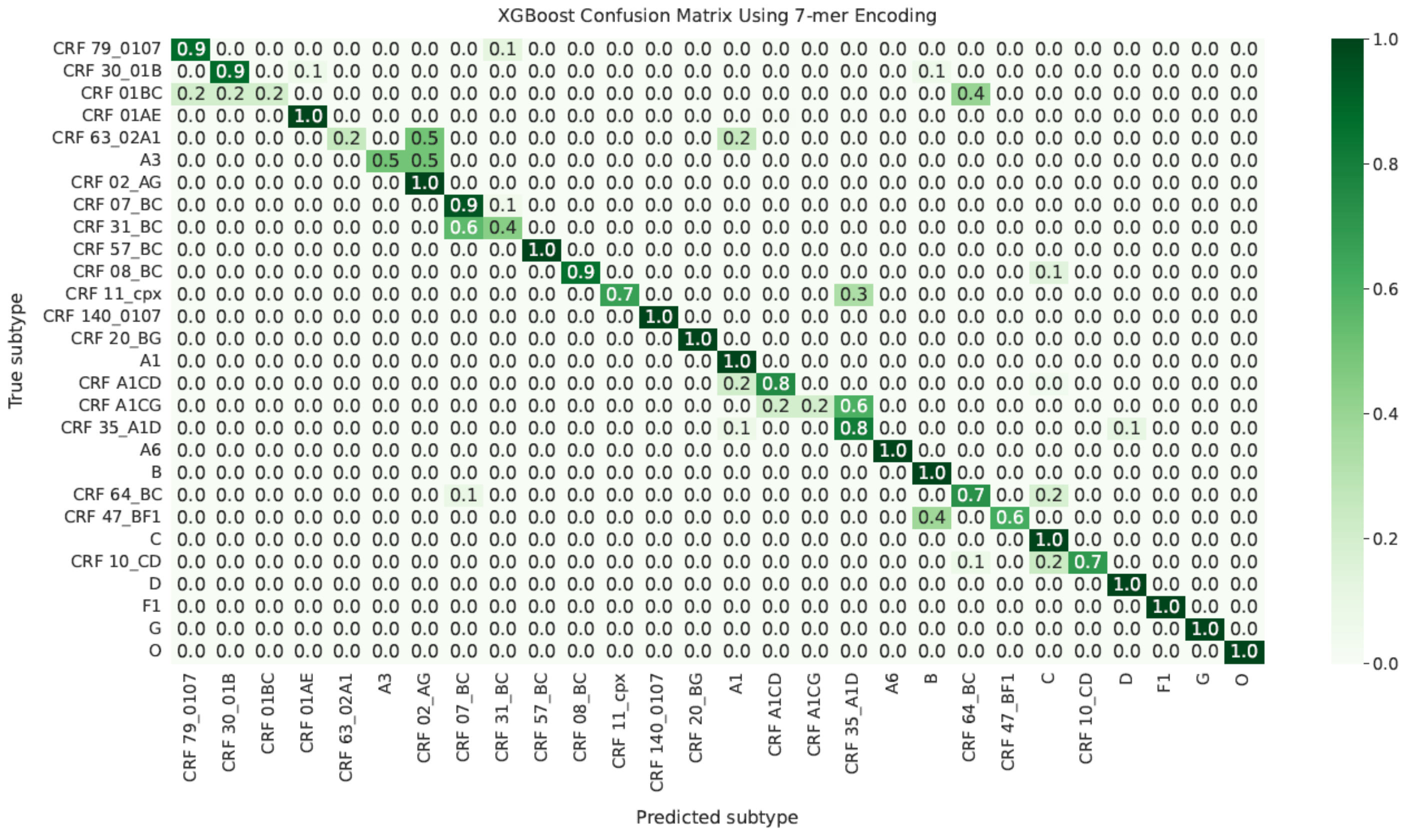

Supplementary Figure 1: Confusion matrix for 7-mer encoding in combination with XGBoost. All values are normalized by class size.
